# Cyclooxygenase-2 mediates gefitinib resistance in non-small cell lung cancer through the EGFR/PI3K/AKT axis

**DOI:** 10.7150/jca.42850

**Published:** 2020-03-26

**Authors:** Qin-fang Deng, Qi-yu Fang, Xian-Xiu Ji, Song-wen Zhou

**Affiliations:** Department of Oncology, Shanghai Pulmonary Hospital, Tongji University School of Medicine, Shanghai, China

**Keywords:** non-small cell lung cancer, gefitinib resistance, COX-2, PI3K-AKT, EGFR

## Abstract

Gefitinib is a potent inhibitor of EGFR and represents the front-line treatment for non-small cell lung cancer (NSCLC) therapeutics. However, NSCLC patients are prone to develop acquired resistance through as yet, undefined mechanisms of resistance. Here, we investigated the role of COX-2 during gefitinib resistance in NSCLC cells and revealed its underlying mechanism(s) of action. We report the upregulation of COX-2 in gefitinib-resistant NSCLC tissues and cells, which is associated with poor prognosis. *In vitro* assays in NSCLC cells (PC9/GR) showed that COX-2 facilitates gefitinib resistance in NSCLC cells through its effects on P-gp, MRP1, and BCRP, and cancer cell migration and invasion. *In vivo*, COX-2 silencing could repress tumor growth. We found that the overexpression of COX-2 enhances the transcription of MMP-2, MMP-7, and MMP-9 which mediates PI3K-AKT activation. In summary, we demonstrate that COX-2 mediates the gefitinib resistance of NSCLC cells through its interaction with EGFR and the PI3K-AKT axis. This highlights COX-2 as a novel molecular target for NSCLC.

## Introduction

Non-small cell lung cancer (NSCLC) is a globally important cancer which remains a major cause of cancer related death despite advances in cancer treatment 1, 2. In recent years, molecularly-targeted therapy has shown great promises in molecular subtypes of lung cancer patients. As an exemplar, EGFR-tyrosine kinase inhibitors (TKI) can prolong progression-free survival for EGFR-mutated lung cancer patients 3, 4. However, the resistance to EGFR-TKI invariably emerged with the treatment 5. Studying the mechanisms of resistance to EGFR-TKI has thus become a research focus in this field. Recent studies showed that EGFR-TKI resistance may be associated with EGFR T790M mutations 6-8, the activation of bypass pathways 9-11, abnormal downstream pathways 12, 13, impairment for EGFR-TKI mediated apoptosis 14,15, histological transformation 16, drug transport 17, and etc. Other resistance mechanisms remain to be found.

Cyclooxygenase-2 (COX-2) regulates inflammation 18. COX-2 plays an important role in carcinogenesis by stimulating growth, survival, invasion, metastasis, and angiogenesis of tumor cells 19-22, 23, 24. EGFR signaling and COX-2 have known interactions in cancer cells 25-27. Young et al showed that COX-2 activation negatively regulates EGFR, and that its overexpression contributes to gefitinib resistance 28.

Here, we show that COX-2 is overexpressed in NSCLC cells that show resistance to gefitinib-therapy. We further demonstrate that COX-2 mediates gefitinib resistance through its interactions with EGFR that ultimately activate PI3K-AKT signaling. These data identify COX-2 as a novel therapeutic target to overcome NSCLC drug resistance.

## Materials and Methods

### Tissues and ethics

The study was approved by our local ethics committee at the Shanghai Pulmonary Hospital, Tongji University School of Medicine. Our study included 32 patients with NSCLC who received gefitinib treatment and developed acquired resistance eventually. NSCLC was diagnosed through pathological assessments. Clinical and demographic data were collected (Table 1). Informed consent was provided by all participants. Enrolled patients provided written informed consent.

### NSCLC cells and culture

The human lung cancer cell lines H1299, H460, SK-MES-1, A549 cells and normal human bronchial epithelial cells were obtained from the Chinese Academy of Sciences). PC9 lung adenocarcinoma cells were from Dr. Takayama (Kyushu University). Gefitinib-resistant PC9/G cells were produced as previously described 29. Briefly, PC9 cells were treated for 24 h with 2.5 μg/ml MNNG followed by treatment with media containing 0.2 μmol/L gefitinib for 7 d. Cells were then grown under normal culture conditions for 14 d. When a substantial number of viable cells were identified, cells were seeded into 96-well plates to obtain single clones from 21 d of culture in the presence of 0.3-0.5 μmol/L gefitinib. Cells were then maintained in 0.05 μmol/L gefitinib under standard culture conditions.

### PI3K assessments

Cells were treated with EGF (ab9697; Abcam) or (ab119139; Abcam). COX-2 was silenced using shRNA approaches (Gene Pharma Co). FLAG-COX2, HA‐EGFR, and the pmyr-AKT were transfected into cells and lentiviruses were isolated. For silencing experiments, the indicated cell lines were infected and PI3K kinase assays were performed as previously described 30.

### RT-qPCR

RNA was TRIzol extracted and cDNA was synthesized. Gene expression was quantified via RT-qPCR and relative gene expression was assessed using the 2^-ΔΔCt^ method and normalized to GAPDH. Primers used for qRT-PCR are shown in Table 2.

### Cell viability assays

Cell viability was assessed in transfected PC9 and PC9/GR cells treated with gefitinib (0-20 μM) for 24 h. Cell viability was assessed in via CCK-8 assays. Absorbances were read at 450 nm. Values were normalized to untreated controls and IC50s were calculated for each treatment.

### Cell migration and invasion assays

For Transwell assays, cells were seeded onto BioCoat Matrigel wells and cell migration and invasiveness were assessed. Migrating/invading cells were quantitated from six microscopic fields. Experiments were performed on 3 or more independent occasions.

### Interaction analysis

NSCLC cells were harvested in RIPA buffer containing protease inhibitor cocktail as previously described 20. Table 3 shows all primary and secondary antibodies used in the study.

### *In vivo* xenograft models

All animal assessments were approved by our internal review board. Male nude mice (n=10, aged 4 weeks, Academy of Military Medical Science, Beijing). Cells in PBS were subcutaneously injected into the flanks to establish tumor models (5×10^6^ cells).

### Statistics

Data were analyzed using SPSS 20.0. Data are the mean ± SD (n=3) compared via a two-tailed Student's t-test or one-way Anova for multiple group comparisons. A Wilcoxon signed rank test was used for the analysis of variance. Mann Whitney U tests were performed as required. Survival analysis was performed using KM analysis. Patient survival was compared via a log-ranked test. Significant values had P-values <0.05.

## Results

### COX-2 is overexpressed in NSCLC tissue and correlates with an unfavorable prognosis

**Figure 1:**
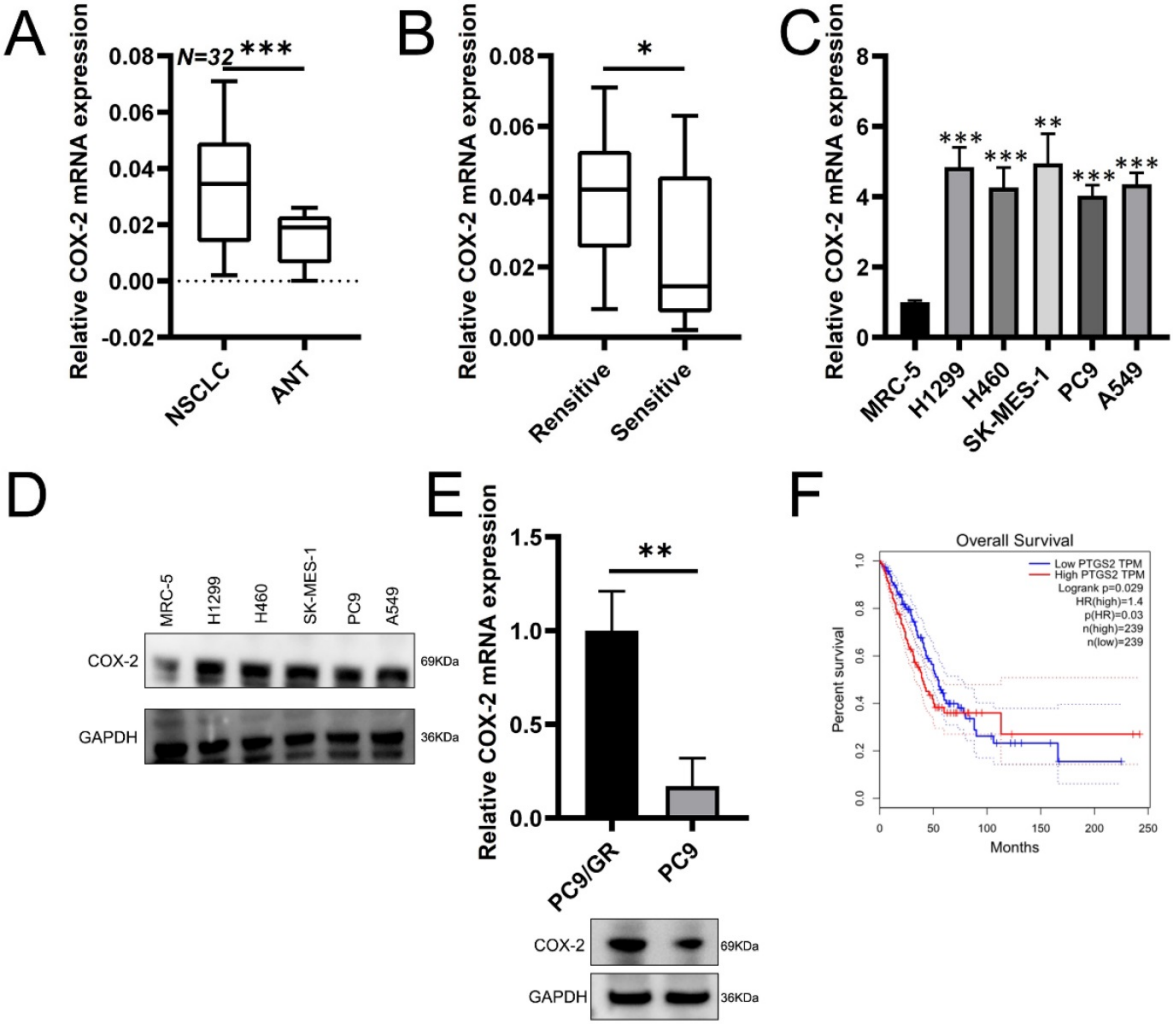
** COX-2 is overexpressed in NSCLC tissue and correlates with poor patient prognosis. (A)** COX-2 mRNA assessed by qRT-PCR analysis in 32 paired NSCLC tissues. **(B) C**OX-2 mRNA levels in NSCLC patients showing gefitinib resistance vs. gefitinib sensitive patients. **(C)** COX-2 mRNA expression in the NSCLC cells vs control cells analyzed by qRT-PCR. (Mean ± SEM, p ≤0.05). **(D)** COX-2 protein expression in NSCLC cells vs. normal cells. **(E)** COX-2 mRNA and protein expression in PC9/GR (gefitinib resistant) and naive PC9 cells. **(F)** OS according to COX-2 expression assessed through the GEPIA. Two-sided log-rank tests were used to determine p-values.

To assess potential role of COX-2 in NSCLC, RT-qPCR was used to assess COX-2 expression in 32 paired NSCLC samples. COX-2 mRNA was highly expressed in NSCLC vs. healthy lung tissue (Figure 1A). In patients resistant to gefitinib, COX-2 mRNA was upregulated vs. gefitinib sensitive patients (Figure 1B). In NSCLC cell lines, COX-2 expression at the protein level also increased (Figure 1C-D). Gefitinib-resistant cells (PC9/GR) also showed higher COX-2 expression than PC9 cells (Figure 1E). GEPIA analysis showed high levels of COX-2 expression in lung adenocarcinoma (LUAD) that correlated with poor patient prognosis (Figure 1F). Thus, COX-2 is overexpressed in NSCLC tissue and is a marker of poor prognosis.

### COX-2 enhances gefitinib resistance and metastasis in NSCLC cells

**Figure 2:**
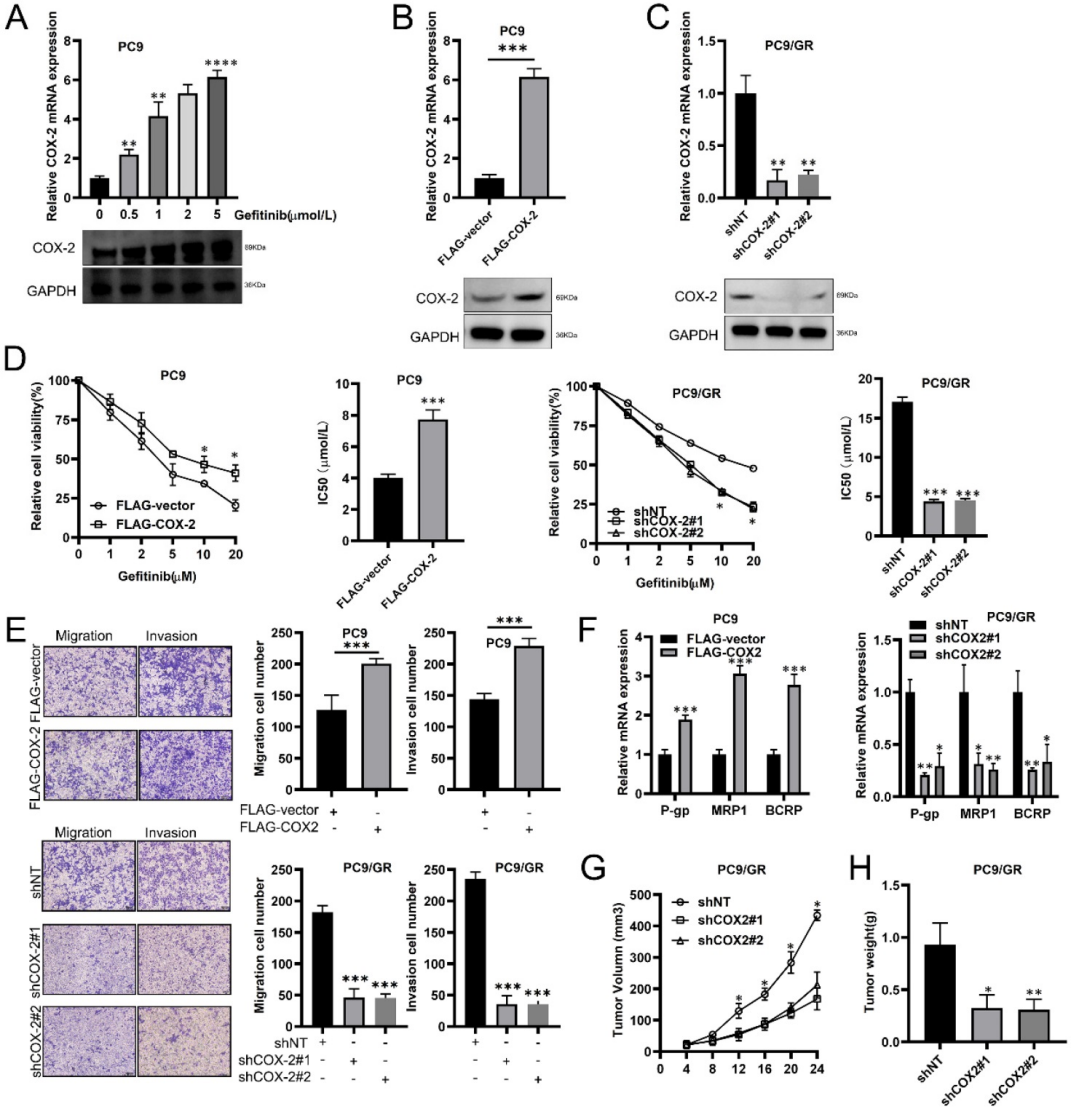
** COX-2 promotes gefitinib chemo resistance and the metastatic phenotypes of NSCLC cells. (A)** RT-qPCR showing COX-2 expression in PC9 cells treated with gefitinib. **(B-C)** PC9 cells overexpressing FLAG-COX-2 or silenced for COX-2 expression (KD, shCOX-2#1, #2) in PC9 cells (PC9/GR). Data are the mean ± SEM. **(D)** Cell viability assessments and IC50 of gefitinib in PC9 vs. PC9/GR cells. Data were compared through ANOVA analysis. **(E)** Transwell assays to show PC9 and PC9/GR cell invasion. Data were compared via a Student's t test. **(F)** P-gp, MRP1, and BCRP expression assessed via RT-qPCR. Data were compared via a Student's t test. **(G-H)** Xenograft assays for the assessment of tumor volume and weight in mice subcutaneously injected with PC9 cells. Data are the mean ± SEM. Data were compared via ANOVA assessments.

We found that in LUAD cells (PC9), COX-2 was upregulated in a concentration dependent manner by gefitinib (Figure 2A). We exogenously overexpressed or silenced COX-2 expression to assess its effects on PC9/GR cells (Figure 2B, 2C). CCK-8 assays showed that the IC50 of gefitinib increased in PC9 cells exogenously expressing COX-2 (Figure 2D), but decreased in COX-2 silenced cells (Figure 2D). Transwell assays showed that the overexpression of COX-2 led to higher levels of cell migration and invasion, whilst the opposite phenotype was observed for COX-2 silenced cells (Figure 2E). RT-qPCR analysis showed that COX-2 overexpression enhanced the levels of P-gp, MRP1, and BCRP, which were inhibited following COX-2 silencing (Figure 2F). In Xenograft mouse models, COX-2 silencing led to lower tumor volumes and weight in those injected with PC9/GR cells (Figure 2G-H). The culmination of these data shows that COX-2 enhances gefitinib resistance, and promotes the metastatic phenotypes of NSCLC cells.

### COX-2 enhances gefitinib resistance and NSCLC metastasis through PI3K-AKT silencing

**Figure 3:**
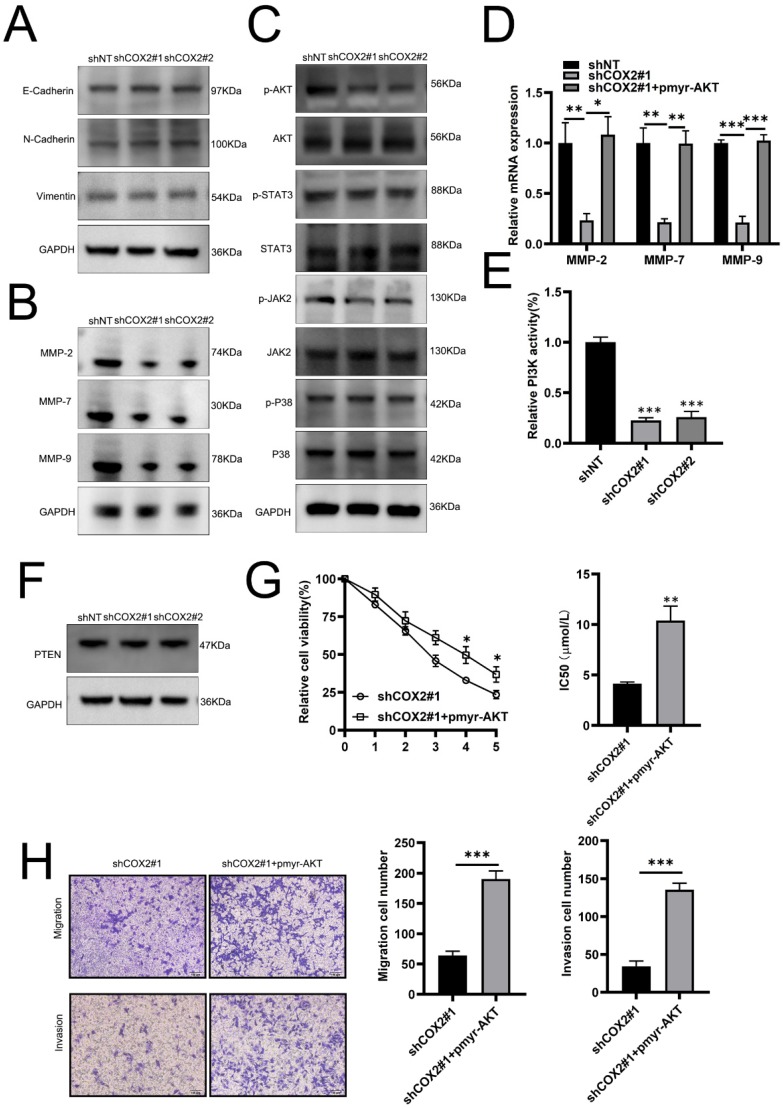
** COX-2 influences the expression of tumor cell MMPs through PI3K-AKT signaling. (A)** Vimentin, E‐cadherin and N‐cadherin expression assessed by WB. **(B)** WB analysis of MMP expression in the indicated cell lines. GAPDH was probed as a control. **(C)** WB assessment of p‐AKT, p-STAT3, p-JAK2, and p‐p38 levels in the indicated cells. GAPDH was probed as a loading control. **(D) Relative** mRNA expression of the indicated MMPs. Data are the mean ± SEM, compared through a Student's t test. **(E)** PI3K activity and **(F)** PTEN expression on the indicated cell lines. GAPDH was probed as a loading control. **(G)** IC50 of gefitinib in PC9/GR cells co-transfected with COX-2 siRNA and/or AKT overexpression plasmids. Data were compared via a one-way ANOVA. **(H)** Migration and invasion assays. Data are the mean ± SEM compared through a Student's t‐test.

To investigate the mechanism(s) through which COX-2 mediates gefitinib resistance, we first examined its role in EMT, a key pathway for cancer metastasis. No notable effects of COX-2 on EMT were observed as vimentin, and N-cadherin expression were largely unchanged between COX-2 overexpressing or silenced lines (Figure 3A), suggesting that COX-2 mediated metastasis and drug resistance in NSCLC cells is EMT independent. Tumor invasion occurs due to the increased proteolytic activity of MMPs that degenerate neighboring stroma, enabling the spread of tumor cells. MMP-2, -7, and -9 expressions were found to decrease in COX-2 silenced cells (Figure 3B). AKT was also inactivated in PC9/ GR cells silenced for COX-2 whilst JNK2, STAT3 and p38 were largely unaffected (Figure 3C). In COX-2 silencedPC9/GR cells, the exogenous expression of AKT significantly increased the expression of MMP-2, -7, and -9 at the mRNA level (Figure 3D). Enhanced PI3K activity was also observed whilst PTEN expression was largely unaffected (Figure 3E, F). The overexpression of AKT led a higher gefitinib IC50 values (Figure 3G). In addition, the migration and invasiveness of NSCLC cells increased following AKT overexpression (Figure 3H). These data highlight the role of PI3K‐AKT signaling in COX-2 mediated gefitinib resistance in NSCLC cells.

### COX-2-binds to EGFR to activate PI3K/AKT signaling

**Figure 4:**
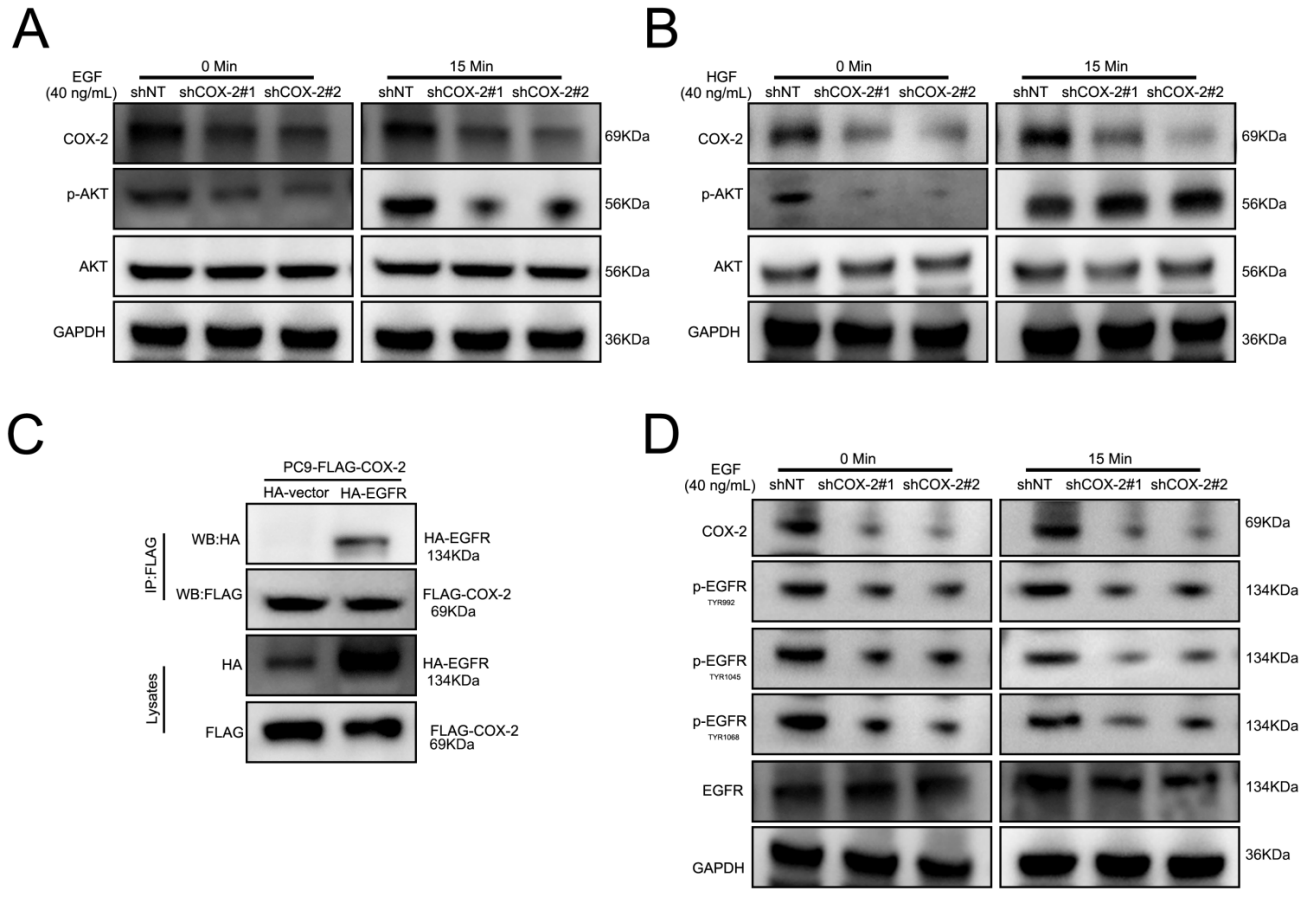
** COX-2-EGFR binding leads to PI3K/AKT activation. (A-B)** Cells were treated with EGF or HGF **(B)** for the indicated time points and WB for the indicated proteins was performed. **(C)** Co-Ip assays of PC9 cells transfected with HA-EGFR, FLAG-COX-2 or empty vector controls. **(D)** Cells were stimulated with EGF exposure for 0 or 15 min and WB analysis was performed as described.

PI3K/AKT signaling activation in response to RTK stimulation. To investigate the ability of COX-2 to activate RTKs in NSCLC cell lines, cells were stimulated with HGF and EGF. As shown in Figure 4A-B, COX-2 silencing prevented EGF stimulated AKT activity in PC9/GR silenced cells vs control shNT cells. In contrast, HGF was unaffected by COX-2 silencing. To investigate a potential interaction between COX-2 and EGFR, FLAG-tagged COX-2 and HA-EGFR were over expressed by transfection in PC9 cells and examined their interaction through co-immunoprecipitation assays. We observed a robust COX-2-EGFR interaction suggestive of its role in COX-2 mediated PI3K signaling (Figure 4C). In addition, upon COX-2 silencing, the phosphorylation of TYR992, TYR1045, and TYR1068 in EGFR decreased in PC9/GR cells (Figure 4D). The culmination of these data highlights COX-2 as a potent oncogene that enhances the targeted resistance of NSCLC cells through its interaction with EGFR, and subsequent activation of the EGFR/PI3K/AKT axis.

## Discussion

Surgical intervention with concomitant chemotherapy remains the front-line treatment for the majority of human cancers 31. The aim of chemotherapy is to eliminate tumor cells following surgical excision of the tumor and to prevent relapse 32. Gefitinib is an EGFR-TKIs that can treat NSCLC patients harboring EGFR mutations 33^.^ Gefitinib is typically administered to patients harboring exon 19 deletions or L858R point mutations 34 and displays high levels of antitumor efficacy 35. In the present study, we found that the exogenous expression of COX-2 in NSCLC cells/tissue enhances their resistance to gefitinib therapy.

COX-2 expression increases in NSCLC cells with gefitinib-induced resistance. The higher levels of COX-2 promote metastatic phenotypes in the tumor cells and promote gefitinib resistance, inferring its role as an oncogene and a key mediator of therapeutic resistance. Resistance to gefitinib through various molecular mechanisms is a major barrier to effective targeted therapy.

To investigate the oncogenic effects of COX-2, we assessed its ability to enhance EMT, a key predictor of cancer metastasis 36-39. Following COX-2 silencing in NSCLC cells, no changes in the expression of known EMT genes were observed. Next, we investigated the effects of COX-2 of an array of known pro-oncogenic signaling pathways. We observed robust AKT activation in COX-2 overexpressing cells and a loss of MMP expression and PI3K signaling upon COX-2 silencing confirming a role for MMPs and AKT in NSCLC metastasis occurrence and development 40-43. We further demonstrated the ability of COX-2 to bind to the EGFR which, in turn, activated EGFR and enhanced PI3K signaling. No such effects were observed for HGF-HGFR signaling in these cells. This revealed new mechanistic insight into the mechanisms through which COX-2 enhances gefitinib resistance, namely through activation of the EGFR/PI3K/AKT axis.

Most solid tumors display enhanced levels of EGFR expression 44-46. For NSCLC, the overexpression of EGFR strongly correlates with the clinical histopathological characteristics of patients. The overexpression of EGFR leads to aggressive tumor invasion and as such, EGFR inhibitors can effectively alleviate the malignant characteristics of many tumor cells 47. For NSCLC, emerging preclinical evidence supports the effectiveness of EGFR inhibition as a therapeutic intervention and treatment 48. Our data highlights that compounds targeting the COX-2-EGFR interaction have potential as future NSCLC therapeutics. The mechanism(s) by which COX-2 regulates PI3K-AKT signaling through its interaction with EGFR now require assessment. We speculate that COX-2 promotes PI3K‐AKT activity through the following mechanisms: 1) COX-2 binds to EGFR to prevent its lysosomal degradation, thus enhancing receptor stability; 2) COX-2 promotes the formation of EGFR dimers to enhance signaling capacity; 3) COX-2 inhibits EGFR endocytosis and the subsequent trafficking of the receptor to the lysosomes for degradation. In future studies, we will explore the mechanisms by which COX-2 enhances PI3K‐AKT signaling mediated through EGFR binding.

In summary, we report, for the first time, that COX-2 mediates the gefitinib-resistant of NSCLC cells. Mechanistically, COX-2 was identified as a novel EGFR interacting partner and enhanced PI3K/AKT signaling through this binding. Taken together, these data highlight the interaction between COX-2 and EGFR and the PI3K/AKT axis as a novel therapeutic strategy to overcome gefitinib resistance in NSCLC cells.

## Figures and Tables

**Table 1:** The Clinicopathological Characteristics of Non-small Cell Lung Cancer Patients

Characteristics	Patients(number)
**Gender**	
Male	22
Female	10
**Age (year)**	
≤60	19
>60	13
**Smoking**	
Yes	15
No	17
**Size of tumor (cm)**	
≤3	20
<3	12
**Differentiation**	
Well/moderate	20
Poor	12
**TNM stage**	
I/II	19
III/IV	13
**Lymph node metastasis**	
N0	18
N1-3	14

TNM: Tumor Node Metastasis

**Table 2:** Sequence of Primers for qRT-PCR

Primer	Sequence (5' to 3')
COX-2 forward primer	TTCCAGATCCAGAGCTCATTAAA
COX-2 reverse primer	CCGGAGCGGGAAGAACT
P-gp forward primer	GATCTTGAAGGGGACCGCAATGGA
P-gp reverse primer	GATGCATAGATCAGCAGGAAAGCAGC
MRP1 forward primer	CTCCCCGGTCTATTCCCATTTCAA
MRP1 reverse primer	TCTCGGTAGCGCAGGCAGTAGTTC
BCRP forward primer	CAGGTGGAGGCAAATCTTCGT
BCRP reverse primer	ACACACCACGGATAAACTGA
MMP-2 forward primer	TCTTGACCAGAATACCATCG
MMP-2 reverse primer	TACTTCACACGGACCACTTG
MMP-7 forward primer	AGTGGTCACCTACAGGATCGTA
MMP-7 reverse primer	ATCTCCTCCGAGACCTGTCC
MMP-9 forward primer	CAACATCACCTATTGGATCC
MMP-9 reverse primer	GGGTGTAGAGTCTCTCGCTG
GAPDH forward primer	TGTGGGCATCAATGGATTTGG
GAPDH reverse primer	ACACCATGTATTCCGGGTCAAT

**Table 3:** The primary antibodies used for WBs and the antibodies used for IP

Antibody	Supplier
COX-2	ab179800, Abcam, 1:1,000
GAPDH	ab181602, Abcam, 1:1,000
vimentin	ab193555, Abcam,1:1,000
E-cadherin	ab194982, Abcam,1:1,000
N-cadherin	ab202030, Abcam,1:1,000
PTEN	ab32119, Abcam, 1:1,000
STAT3	ab119352, Abcam,1:1,000
JAK2	ab108596, Abcam,1:1,000
total p38(T-p38)	ab31828, Abcam,1:1,000
total Akt (T-Akt)	ab179463, Abcam,1:1,000
phosphorylated EGFR (p-EGFR, at Y992)	ab81440, Abcam, 1:1,000
phosphorylated EGFR (p-EGFR, at Y1045)	ab24928, Abcam, 1:1,000
phosphorylated EGFR (p-EGFR, at Y1068)	ab40815, Abcam, 1:1,000
phosphorylated p38(p-p38)	ab4822, Abcam, 1:1,000
phosphorylated STAT3(p-STAT3)	ab76315, Abcam, 1:1,000
phosphorylated JAK2(p-JAK2)	Ab32101, Abcam,1:000
phosphorylated Akt (p-Akt)	ab38449, Abcam, 1:1,000
HA-probe	sc-2362, Santa Cruz Biotechnology; 1:800
FLAG-probe	F1804, Sigma-Aldrich; 1:2,000
EGFR	ab52894, Abcam, 1:1,000
FLAG M2-affinity gel	A2220, Sigma-Aldrich; 20 ml per reaction

## References

[1] Torre LA, Bray F, Siegel RL (2015). Global cancer statistics, 2012. CA Cancer J Clin.

[2] Siegel RL, Miller KD, Jemal A (2018). 0000-0002-0000-4111 AO: Cancer statistics, 2018. CA Cancer J Clin.

[3] Mok TS, Wu YL, Thongprasert S (2009). Gefitinib or carboplatin-paclitaxel in pulmonary adenocarcinoma. N Engl J Med.

[4] Maemondo M, Inoue A, Kobayashi K (2010). Gefitinib or chemotherapy for non-small-cell lung cancer with mutated EGFR. N Engl J Med.

[5] Kobayashi S, Boggon TJ, Dayaram T (2005). EGFR mutation and resistance of non-small-cell lung cancer to gefitinib. N Engl J Med.

[6] Yu HA, Arcila ME, Rekhtman N (2013). Analysis of tumor specimens at the time of acquired resistance to EGFR-TKI therapy in 155 patients with EGFR-mutant lung cancers. Clin Cancer Res.

[7] Yun CH, Mengwasser KE, Toms AV (2008). The T790M mutation in EGFR kinase causes drug resistance by increasing the affinity for ATP. Proc Natl Acad Sci U S A.

[8] Fujita Y, Suda K, Kimura H (2012). Highly sensitive detection of EGFR T790M mutation using colony hybridization predicts favorable prognosis of patients with lung cancer harboring activating EGFR mutation. J Thorac Oncol.

[9] Nakata S, Tanaka H, Ito Y (2014). Deficient HER3 expression in poorly-differentiated colorectal cancer cells enhances gefitinib sensitivity. Int J Oncol.

[10] Engelman JA, Zejnullahu K, Mitsudomi T (2007). MET amplification leads to gefitinib resistance in lung cancer by activating ERBB3 signaling. Science.

[11] Ju L, Zhou C (2013). Association of integrin beta1 and c-MET in mediating EGFR TKI gefitinib resistance in non-small cell lung cancer. Cancer Cell Int.

[12] Sos ML, Koker M, Weir BA (2009). PTEN loss contributes to erlotinib resistance in EGFR-mutant lung cancer by activation of Akt and EGFR. Cancer Res.

[13] Tarantini A, Maitre A, Lefebvre E (2011). Polycyclic aromatic hydrocarbons in binary mixtures modulate the efficiency of benzo[a]pyrene to form DNA adducts in human cells. Toxicology.

[14] Ng KP, Hillmer AM, Chuah CT (2012). A common BIM deletion polymorphism mediates intrinsic resistance and inferior responses to tyrosine kinase inhibitors in cancer. Nat Med.

[15] Kiyota M, Kuroda J, Yamamoto-Sugitani M (2013). FTY720 induces apoptosis of chronic myelogenous leukemia cells via dual activation of BIM and BID and overcomes various types of resistance to tyrosine kinase inhibitors. Apoptosis.

[16] Wilson C, Nicholes K, Bustos D (2014). Overcoming EMT-associated resistance to anti-cancer drugs via Src/FAK pathway inhibition. Oncotarget.

[17] Sodani K, Tiwari AK, Singh S (2012). GW583340 and GW2974, human EGFR and HER-2 inhibitors, reverse ABCG2- and ABCB1-mediated drug resistance. Biochem Pharmacol.

[18] Smith WL, DeWitt DL, Garavito RM (2000). Cyclooxygenases: structural, cellular, and molecular biology. Annu Rev Biochem.

[19] Yang L, Amann JM, Kikuchi T (2007). Inhibition of epidermal growth factor receptor signaling elevates 15-hydroxyprostaglandin dehydrogenase in non-small-cell lung cancer. Cancer Res.

[20] Singh B, Berry JA, Shoher A (2007). COX-2 involvement in breast cancer metastasis to bone. Oncogene.

[21] Chang YW, Marlin JW, Chance TW (2006). RhoA mediates cyclooxygenase-2 signaling to disrupt the formation of adherens junctions and increase cell motility. Cancer Res.

[22] Grosch S, Maier TJ, Schiffmann S (2006). Cyclooxygenase-2 independent anticarcinogenic effects of selective COX-2 inhibitors. J Natl Cancer Inst.

[23] Tsai WC, Tsai ST, Jin YT (2006). Cyclooxygenase-2 is involved in S100A2-mediated tumor suppression in squamous cell carcinoma. Mol Cancer Res.

[24] Watwe V, Javle M, Lawrence D (2005). Cyclooxygenase-2 levels before and after chemotherapy: a study in rectal cancer. Am J Clin Oncol.

[25] Dannenberg AJ, Lippman SM, Mann JR (2005). Cyclooxygenase-2 and epidermal growth factor receptor: pharmacologic targets for chemoprevention. J Clin Oncol.

[26] Half E, Sun Y, Sinicrope FA (2007). Anti-EGFR and ErbB-2 antibodies attenuate cyclooxygenase-2 expression and cooperatively inhibit survival of human colon cancer cells. Cancer Lett.

[27] Lanza-Jacoby S, Burd R, Rosato FE Jr (2006). Effect of simultaneous inhibition of epidermal growth factor receptor and cyclooxygenase-2 in HER-2/neu-positive breast cancer. Clin Cancer Res.

[28] Kim YM, Park SY, Pyo H (2009). Cyclooxygenase-2 negatively regulates expression of epidermal growth factor receptor and causes resistance to gefitinib in COX-2-overexpressing cancer cells. Mol Cancer Res.

[29] Kim YM, Park SY, Pyo H (2009). Cyclooxygenase-2 negatively regulates expression of epidermal growth factor receptor and causes resistance to gefitinib in COX-2-overexpressing cancer cells. Mol Cancer Res.

[30] Wen W, Ding J, Sun W (2012). Cyclin G1-mediated epithelial-mesenchymal transition via phosphoinositide 3-kinase/Akt signaling facilitates liver cancer progression. Hepatology.

[31] Lin G, Li C, Huang C (2018). Co-expression of NF-kappaB-p65 and phosphorylated NF-kappaB-p105 is associated with poor prognosis in surgically resectable non-small cell lung cancer. J Cell Mol Med.

[32] Chen J, Sheng X, Ma H (2018). WDR79 mediates the proliferation of non-small cell lung cancer cells by regulating the stability of UHRF1. J Cell Mol Med.

[33] Su J, Zhong W, Zhang X (2017). Molecular characteristics and clinical outcomes of EGFR exon 19 indel subtypes to EGFR TKIs in NSCLC patients. Oncotarget.

[34] Rawluk J, Waller CF (2018). Gefitinib. Recent Results Cancer Res.

[35] Zhang W, Wei Y, AUID- Oho (2018). Gefitinib provides similar effectiveness and improved safety than erlotinib for east Asian populations with advanced non-small cell lung cancer: a meta-analysis. BMC Cancer.

[36] Brabletz T (2012). EMT and MET in metastasis: where are the cancer stem cells. Cancer Cell.

[37] Zheng H, Shen M, Zha YL (2014). PKD1 phosphorylation-dependent degradation of SNAIL by SCF-FBXO11 regulates epithelial-mesenchymal transition and metastasis. Cancer Cell.

[38] Mak P, Leav I, Pursell B (2010). ERbeta impedes prostate cancer EMT by destabilizing HIF-1alpha and inhibiting VEGF-mediated snail nuclear localization: implications for Gleason grading. Cancer Cell.

[39] Singh A, Greninger P, Rhodes D (2009). A gene expression signature associated with "K-Ras addiction" reveals regulators of EMT and tumor cell survival. Cancer Cell.

[40] Perez-Ramirez C, Canadas-Garre M, Molina MA (2015). PTEN and PI3K/AKT in non-small-cell lung cancer. Pharmacogenomics.

[41] Wang X, Xu J, Chen J (2019). IL-22 Confers EGFR-TKI Resistance in NSCLC via the AKT and ERK Signaling Pathways. Front Oncol.

[42] Khan P, Bhattacharya A, Sengupta D (2019). Aspirin enhances cisplatin sensitivity of resistant non-small cell lung carcinoma stem-like cells by targeting mTOR-Akt axis to repress migration. Sci Rep.

[43] Liao Z, Zheng Q, Wei T (2019). MicroRNA-561 affects proliferation and cell cycle transition through PTEN/AKT signaling pathway by targeting P-REX2a in NSCLC. Oncol Res.

[44] Kondapaka SB, Fridman R, Reddy KB (1997). Epidermal growth factor and amphiregulin up-regulate matrix metalloproteinase-9 in human breast cancer cells. Int J Cancer.

[45] Fujino S, Enokibori T, Tezuka N (1996). A comparison of epidermal growth factor receptor levels and other prognostic parameters in non-small cell lung cancer. Eur J Cancer.

[46] Herbst RS, Langer CJ (2002). Epidermal growth factor receptors as a target for cancer treatment: the emerging role of IMC-C225 in the treatment of lung and head and neck cancers. Semin Oncol.

[47] Giaccone G (2005). Targeting HER1/EGFR in cancer therapy: experience with erlotinib. Future Oncol.

[48] Lee DH (2017). Treatments for EGFR-mutant non-small cell lung cancer: The road to a success, paved with failures. Pharmacol Ther.

